# Effect of Mg^2+^ on Enhancing Stabilization and Microwave Absorption Performance of Mg_x_Fe_3−x_O_4_

**DOI:** 10.3390/molecules30224418

**Published:** 2025-11-15

**Authors:** Yu Du, Jianning Sun, Bin Li, Xueyan Du, Yongkun Yang, Xiaoming Li, Xingmin Guo

**Affiliations:** 1 State Key Laboratory of Advanced Metallurgy and School of Metallurgical and Ecological Engineering, University of Science and Technology Beijing, Beijing 100083, China; yudu930413@163.com (Y.D.); b20200109@xs.ustb.edu.cn (J.S.); 2 School of Metallurgy and Environment and State Key Laboratory of Advanced Processing and Recycling of Non-Ferrous Metals, Lanzhou University of Technology, Lanzhou 730050, China; libin@lut.edu.cn (B.L.); duxy@lut.edu.cn (X.D.); 3 School of Metallurgical Engineering, Xi’an University of Architecture and Technology, Xi’an 710055, China; yangyk@xauat.edu.cn (Y.Y.); lixm@xauat.edu.cn (X.L.)

**Keywords:** magnetite, Mg^2+^, solid solution, reflection loss, impedance matching

## Abstract

Magnetite (Fe_3_O_4_) is an essential material for enhancing microwave absorption performance and is widespread and abundant as a solid solution in natural minerals and metallurgical slags. In this work, the effect of Mg^2+^ on the structure, stabilization, and microwave absorption performance of magnesium-containing magnetite (Mg_x_Fe_3−x_O_4_) was investigated. On the basis of experiments on the reactions of Fe_2_O_3_ and MgO under different levels of *p*_CO_/(*p*_CO_ + *p*_CO2_), Mg_x_Fe_3−x_O_4 (x=0.0,0.2,0.4,0.6,1.0)_ was synthesized, and Mg^2+^ was found to inhibit the re-oxidation of magnetite. On this basis, the microwave absorption performance of various synthesized Mg_x_Fe_3−x_O_4_ samples was measured and analyzed, where Mg^2+^ was found to enhance the microwave absorption performance of Fe_3_O_4_, and the RL_min_ value of Mg_0.2_Fe_2.8_O_4_ increased to −50.43 dB compared to that of −19.20 dB for Fe_3_O_4_. Furthermore, the enhancement mechanism of Mg^2+^ was revealed through impedance matching, dielectric and magnetic loss tangents, and magnetization curves, where the Mg^2+^ ions were found to accelerate the hopping of electrons and change the impedance matching of Mg_x_Fe_3−x_O_4_ to a more ideal state.

## 1. Introduction

The proliferation of 5G technology has propelled the application of electromagnetic waves to an unprecedented height [1,2]. While electromagnetic waves have brought much convenience to humanity, they have also introduced the increasingly prominent issues of electromagnetic radiation and electromagnetic interference (EMI) [3]. In this context, effectively reducing the impact of electromagnetic fields on device performance and addressing their potential human health effects has become of paramount importance. Consequently, research into electromagnetic-wave-absorbing materials, particularly the design, fabrication, and application of high-performance electromagnetic-wave-absorption materials, has emerged as a key focus in current studies [4,5].

Magnetite, with the primary chemical composition of Fe_3_O_4_, possesses a series of essential parameters for enhancing microwave absorption, including complex permeability, complex permittivity, high saturation magnetization, and a high Curie temperature [6,7,8,9,10]. This material offers advantages such as ease of fabrication, good biocompatibility, superparamagnetic properties, and favorable chemical stability. Furthermore, it exhibits significant magnetic loss under alternating electromagnetic fields. It has been verified that Fe_3_O_4_ crystal features a cubic inverse spinel structure, where oxygen (O) atoms form a face-centered cubic (fcc) close-packed arrangement, and the trivalent iron cations (Fe^3+^) occupy the tetrahedral interstitial sites (A-sites), whereas the octahedral interstitial sites (B-sites) are occupied by two Fe^3+^ cations and one Fe^2+^ cation. At room temperature, electron hopping occurs between the Fe^3+^ and Fe^2+^ cations at the B-sites, endowing magnetite with high electrical conductivity. Consequently, Fe_3_O_4_-based materials can simultaneously generate both dielectric loss and magnetic loss upon interaction with microwaves, resulting in effective wave absorption performance.

Today, some metallurgical ores and slag by-products containing relatively large amounts of magnetite, including nickel slag [11], steel slag [12], copper slag [13,14], titanium slag [15], and vanadium slag [16], have been utilized to prepare construction or building materials that possess microwave absorption properties. In addition to the mechanical strength of each individual mineral and slag product, the enhancement effect of magnetite on microwave absorption is an essential factor for enabling the higher-value application of various types of slag. In natural minerals, magnetite mainly exists in ore in the form of a solid solution [17,18]. Especially in some metallurgical ores and slags, where the bivalent ions of some gangue elements (Mg^2+^, Ni^2+^, Cu^2+^, et al.) tend to react with stoichiometric magnetite to replace the Fe^2+^ to form a non-stoichiometric M^2+^-doping magnetite solid solution. However, up to now, the definite structures, properties, and corresponding applications of these solid solutions in ores or slag were still an enormous challenge for the researchers, due to the lack of a database for the corresponding non-stoichiometric compounds. This would greatly inhibit the application of various ores or slags. Furthermore, the doping ions are confirmed to possess the ability to improve the dielectric polarization of the materials in some previous studies [19,20,21].

Among various slag by-products, magnesium ions are usually the most abundant and are the easiest to react with magnetite to form magnesium-containing magnetite solid solutions [22,23,24]. In this study, the effect of Mg^2+^ on the stabilization and microwave absorption properties of magnetite was investigated. Through the solid reaction experiments of MgO with Fe_2_O_3_, the single magnesium-containing magnetite solid solutions (Mg_x_Fe_3−x_O_4_) were synthesized, and the effect of Mg^2+^ on the structure of Mg_x_Fe_3−x_O_4_ was clarified. On the base, the microwave absorption properties, including reflection loss, dielectric and magnetic loss, electromagnetic performance, and impedance matching of Mg_x_Fe_3−x_O_4_ were tested and analyzed, and the enhancement mechanism of Mg^2+^ on the microwave absorption properties of magnetite was also revealed. This work aims to provide a new method for the performance research of solid solutions in metallurgical slags, to broaden the application field of metallurgical slag as a construction or building material.

## 2. Results and Discussion

### 2.1. Effect of Mg^2+^ on the Formation of Mg_x_Fe_3−x_O_4_

**Figure 1:**
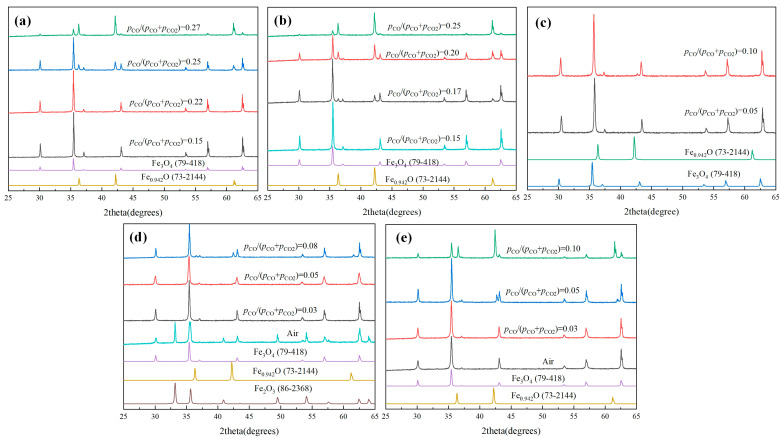
XRD patterns of different Mg_x_Fe_3−x_O_4_ samples sintered at 900 °C for 2 h under different *p*_CO_/(*p*_CO_ + *p*_CO2_): (**a**) x = 0.0, (**b**) x = 0.2, (**c**) x = 0.4, (**d**) x = 0.6, (**e**) x = 1.0.

XRD patterns of different Mg-doping magnetite samples sintered at 900 °C for 2 h under different *p*_CO_/(*p*_CO_ + *p*_CO2_) are shown in Figure 1. As shown in Figure 1a, for the sample of x = 0.0, when the *p*_CO_/(*p*_CO_ + *p*_CO2_) was 0.15, all of the diffraction peaks were matched well with Fe_3_O_4_ (PDF#79-418). When the *p*_CO_/(*p*_CO_ + *p*_CO2_) increased to 0.22, another kind of diffraction peak appeared, which was matched with Fe_0.942_O (PDF#73-2144). With the increase of *p*_CO_/(*p*_CO_ + *p*_CO2_), the diffraction peak intensity of Fe_0.942_O was further increased, indicating that increasing *p*_CO_/(*p*_CO_ + *p*_CO2_) was beneficial for the formation of Fe_0.942_O. While, as shown in Figure 1b, recording to Mg_0.2_Fe_2.8_O_4_, as the *p*_CO_/(*p*_CO_ + *p*_CO2_) was increased to 0.17, the diffraction peaks of Fe_0.942_O appeared. Furthermore, compared with the XRD results of x = 0, there was a significant increase in the peak intensity of Fe_0.942_O under the same atmosphere, such as *p*_CO_/(*p*_CO_ + *p*_CO2_) of 0.22 and 0.25. Furthermore, the *p*_CO_/(*p*_CO_ + *p*_CO2_) for the appearance of Fe_0.942_O became 0.08 and 0.05, respectively, as shown in Figure 1d,e, recording to Mg_0.6_Fe_2.4_O_4_ and MgFe_2_O_4_. On the other hand, it can be seen that some hematite phase could appear under the air atmosphere in the sample of x = 0.6. On the contrary, there was no hematite phase in the sample of x = 1.0. It was obvious that the doping of Mg^2+^ in magnetite not only could inhibit the re-oxidation of magnetite, but also could promote the reduction of Mg_x_Fe_3−x_O_4_ to Mg_x_Fe_1−x_O, where the *p*_CO_/(*p*_CO_ + *p*_CO2_) of reduction beginning for Fe_3_O_4_, Mg_0.2_Fe_2.8_O_4_, Mg_0.4_Fe_2.6_O_4_, Mg_0.6_Fe_2.4_O_4,_ and MgFe_2_O_4_ varied from 0.22, 0.17, 0.10, 0.08, and 0.05, respectively. Furthermore, the results also indicate that the doping of Mg^2+^ in magnetite was also beneficial for the stabilization of magnetite in the air or oxidizing atmosphere, which was of great significance for utilization in the area of building materials or coatings.

**Figure 2:**
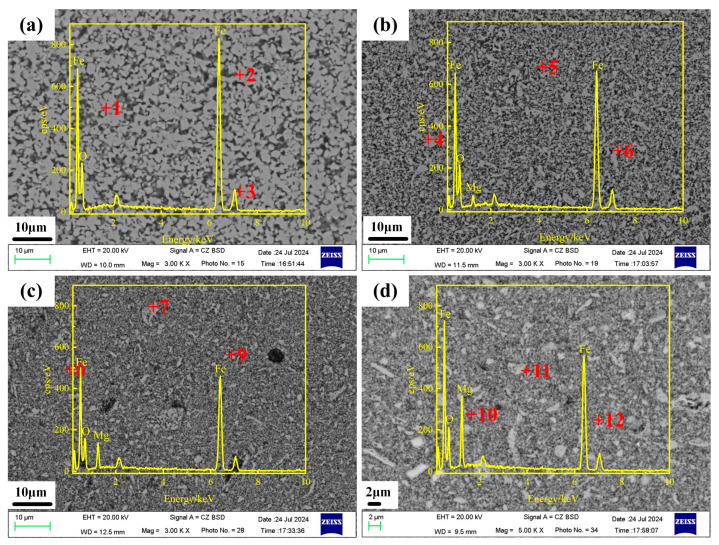
SEM images of synthesized Mg_x_Fe_3−x_O_4_ samples (**a**) x = 0.0, *p*_CO_/(*p*_CO_ + *p*_CO2_) = 0.15; (**b**) x = 0.2, *p*_CO_/(*p*_CO_ + *p*_CO2_) = 0.15; (**c**) x = 0.6, *p*_CO_/(*p*_CO_ + *p*_CO2_) = 0.03 (**d**) x = 1.0, air.

Based on the XRD patterns of different Mg_x_Fe_3−x_O_4_ samples as above, the formation conditions of single Mg_x_Fe_3−x_O_4_ were obtained, and the corresponding SEM images of synthesized Fe_3_O_4_, Mg_0.2_Fe_2.8_O_4_, Mg_0.6_Fe_2.4_O_4,_ and MgFe_2_O_4_ are shown in Figure 2. It can be seen that all four Mg^2+^-doping magnetites possessed a uniform state in a microscopic field of view, indicating single uniform Mg_x_Fe_3−x_O_4_ solid solutions have been synthesized through the reaction of Fe_2_O_3_ and MgO. Furthermore, the element distribution of synthesized Mg_x_Fe_3−x_O_4_ solid solutions was also investigated by the method of EDS, and the corresponding results are presented in Table 1. From the atomic percentage of Fe, Mg, and O at the three positions for the four Mg_x_Fe_3−x_O_4_ solid solutions, it was found that the variation of Fe, Mg, and O was small. Moreover, from the calculated average data as presented in Table 1, the average data for Fe, Mg, and O were close to the chemical formula of Fe_3_O_4_, Mg_0.2_Fe_2.8_O_4_, Mg_0.6_Fe_2.4_O_4,_ and MgFe_2_O_4_, respectively. This further confirmed that the distribution of Mg^2+^ in the synthesized Mg_x_Fe_3−x_O_4_ solid solutions was basically homogeneous, which provides a guarantee for the investigation of the effect of Mg^2+^ on the microwave absorption properties of magnetite.

**Figure 3:**
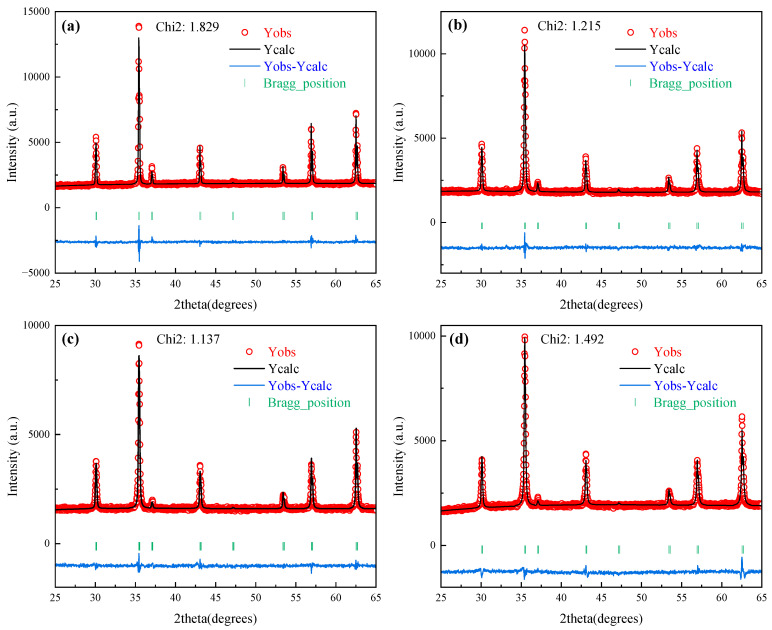
Rietveld refinement results of synthesized Mg_x_Fe_3−x_O_4_: (**a**) x = 0.0, (**b**) x = 0.2, (**c**) x = 0.6, (**d**) x = 1.0.

**Figure 4:**
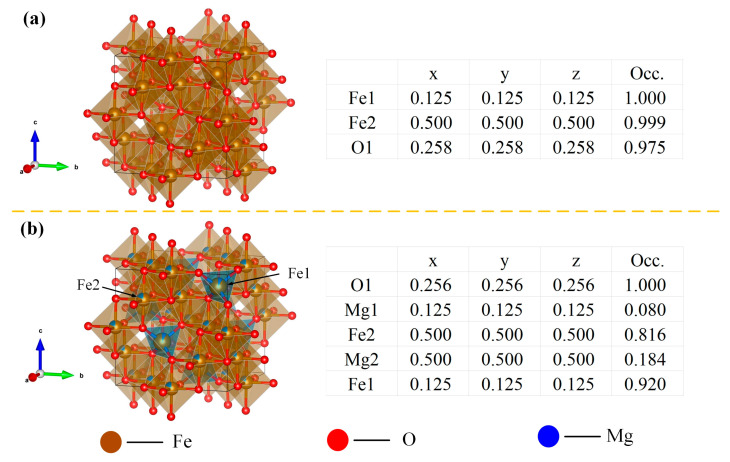
Schematic diagram and structure parameters of synthesized Mg_x_Fe_3−x_O_4_: (**a**) x = 0.0, (**b**) x = 0.6.

Due to the deficiency of systematic PDF cards for non-stoichiometric Mg_x_Fe_3−x_O_4_ solid solutions in the current XRD database, the XRD patterns of synthesized Mg_x_Fe_3−x_O_4_ were refined, and the Rietveld refinement results, final refined reliability factors, and cell parameters of synthesized Mg_x_Fe_3−x_O_4_ are shown in Figure 3 and Table 2. The reliability factor value (Chi2) for all of Mg_x_Fe_3−x_O_4_ was below 2, which demonstrated that the fitting refinement results for synthesized Mg_x_Fe_3−x_O_4_ are ideal. Furthermore, as can be seen from the structure parameters from Table 2, the synthesized Mg_x_Fe_3−x_O_4_ were all existed as a cubic space group with similar cell parameters (a = ~8.39 Å) and the same bond angle (α = β = γ = 90°), which indicated that the doping of Mg^2+^ in magnetite would not change the basic crystal structure. Figure 4a,b present the schematic diagram and structure parameters of the synthesized Fe_3_O_4_ and Mg_0.6_Fe_2.4_O_4_. It can be seen that when the Mg^2+^ was incorporated into the crystal lattice of Fe_3_O_4_, the Mg^2+^ ions could both replace the tetrahedron (A-site) and octahedron (B-site) of magnetite.

### 2.2. Microwave Absorption Performance of Mg_x_Fe_3−x_O_4_

**Figure 5:**
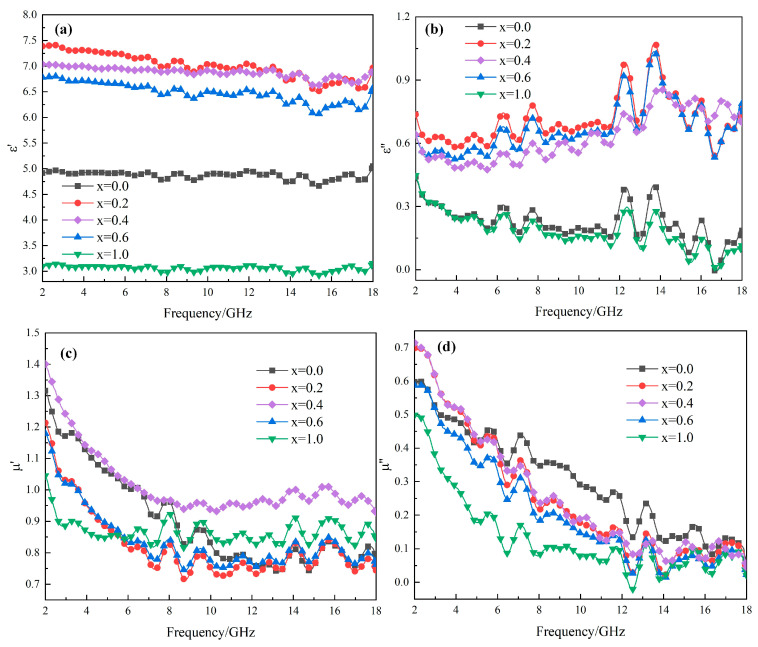
Frequency dependences of permittivity and permeability of Mg_x_Fe_3−x_O_4_: (**a**) real part of permittivity; (**b**) imaginary part of permittivity; (**c**) real part of permeability; (**d**) imaginary part of permeability.

Figure 5 presents the permittivity and permeability values of Mg_x_Fe_3−x_O_4_ as a function of frequency, where the real part and imaginary part of permittivity, real part and imaginary part of permeability were shown in Figure 5a–d, respectively. It was obvious that for all the permittivity or permeability parts of Mg_x_Fe_3−x_O_4_, it showed a similar tendency as a function of frequency, indicating that the basic structure and properties of magnetite were not changed a lot. For the permittivity of Mg_x_Fe_3−x_O_4,_ as shown in Figure 5a,b, it can be seen that the permittivity values for solid solutions including Mg_0.2_Fe_2.8_O_4_, Mg_0.4_Fe_2.6_O_4,_ and Mg_0.6_Fe_2.4_O_4_ were far higher than those for stoichiometric Fe_3_O_4_ and MgFe_2_O_4_, indicating that an increase in electrical energy storage and consumption appeared after Mg^2+^ was doped into Fe_3_O_4_. For permeability parts of Mg_x_Fe_3−x_O_4,_ as shown in Figure 5c,d, it showed a decreasing trend was observed as a function of frequency.

**Figure 6:**
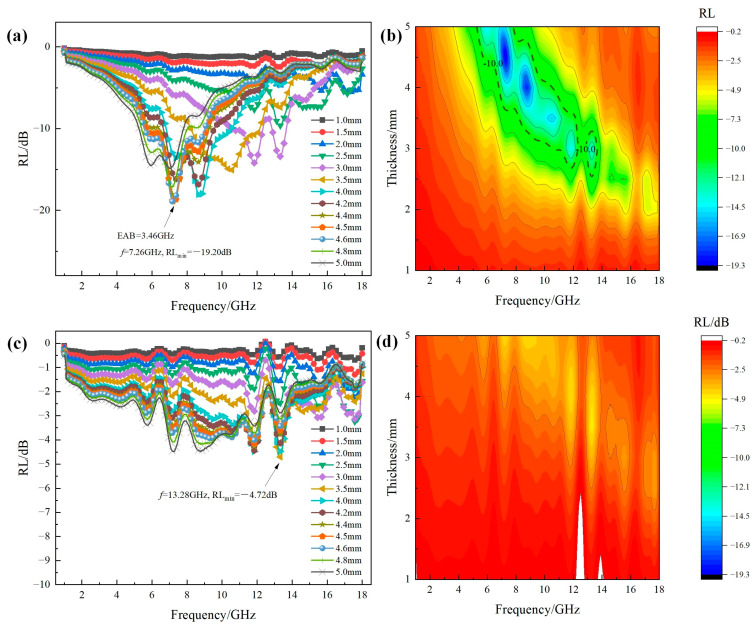
(**a**,**c**) RL-frequency curves and (**b**,**d**) 2D contour RL maps at different thicknesses of Fe_3_O_4_ and MgFe_2_O_4_ in the frequency range of 1–18 GHz: (**a**,**b**) Fe_3_O_4_; (**c**,**d**) MgFe_2_O_4._

Figure 6 shows the RL-frequency curves and 2D contour RL maps of Fe_3_O_4_ and MgFe_2_O_4_ at different thicknesses (1–5 mm) in the frequency range of 1–18 GHz. It can be seen that the two stoichiometric compounds exhibited totally different microwave absorption properties. For the RL curves of Fe_3_O_4_ as shown in Figure 6a, except for the thickness of 1–2.5 mm, all of the other thicknesses possessed the EAB range (RL < −10 dB). Furthermore, it was obvious that with the increase in thickness, the EAB range was gradually moving towards a lower frequency direction. For a thickness of 4.5 mm, the RL_min_ value has reached −19.20 dB at the frequency of 7.26 GHz. However, for the RL curves of MgFe_2_O_4_ as shown in Figure 6c, the thickness of 3.5 mm possessed the RL_min_ peak of −4.72 dB at the frequency of 13.28 GHz. From the 2D contour RL maps as the functions of thickness and frequency, as shown in Figure 6b,d, it can be visually seen that the difference of absorption properties between Fe_3_O_4_ and MgFe_2_O_4_, where Fe_3_O_4_ possessed a relatively large area of EAB range, inversely, there was no EAB range for MgFe_2_O_4_.

**Figure 7:**
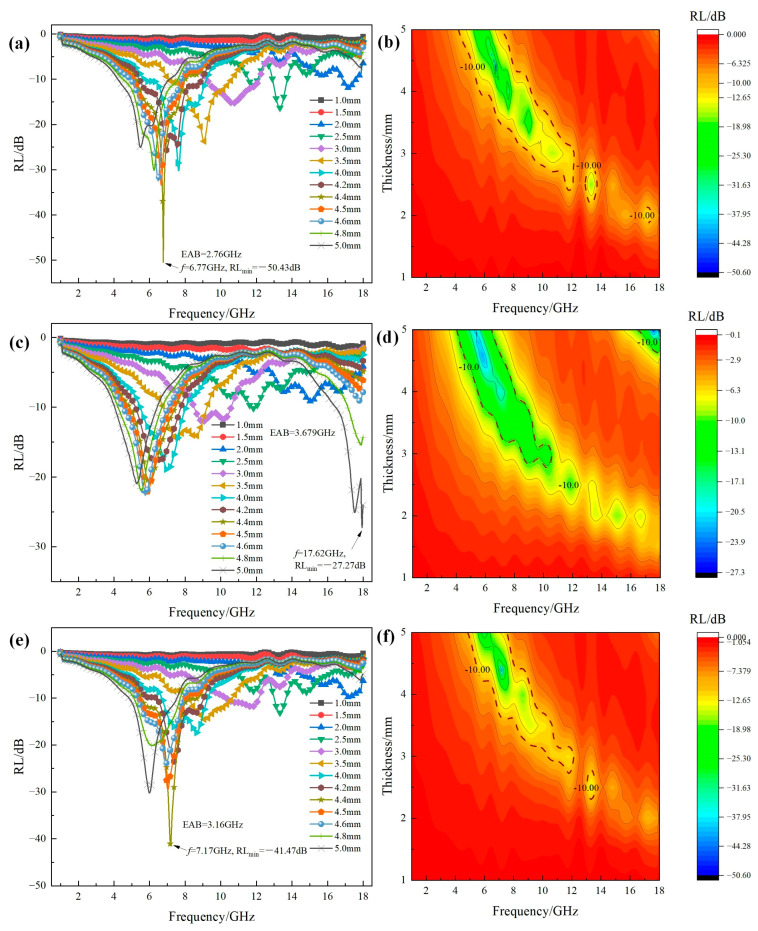
(**a**,**c**,**e**) RL-frequency curves and (**b**,**d**,**f**) 2D contour RL maps at different thicknesses of Mg_x_Fe_3−x_O_4_ solid solution: (**a**,**b**) x = 0.2; (**c**,**d**) x = 0.4; (**e**,**f**) x = 0.6.

RL-frequency curves and 2D contour RL maps at different thicknesses of Mg_x_Fe_3−x_O_4_ solid solution are shown in Figure 7. It can be seen that after Mg^2+^ has been doped into the magnetite, the microwave absorption properties were further enhanced compared to the single Fe_3_O_4_ and MgFe_2_O_4_. As shown in Figure 7a, for the reflection curves of Mg_0.2_Fe_2.8_O_4_, it can be seen that RL_min_ peak values for the thickness of 3–5 mm were further decreased to −25–−50 dB in the frequency range of 2–10 GHz compared to single Fe_3_O_4_, and the RL_min_ value of 4.4 mm has reached −50.43 dB. This confirmed that the Mg_x_Fe_3−x_O_4_ possessed the potential for application in the field of microwave absorbing coating [6]. As shown in Figure 7b, it can be seen that the area of EAB in 2D contour RL maps was significantly increased compared with single Fe_3_O_4_, especially in the lower thickness and frequency range. When the doping content of Mg^2+^ in the magnetite was increased to x = 0.4, as shown in Figure 7c,d, the RL value showed a slight decrease to −27.27 dB, and the area of EAB in the 2D contour RL map also showed a slight increase, especially in the higher frequency range. When the doping content of Mg^2+^ was increased to x = 0.6, as shown in Figure 7e,f, the RL_min_ value showed a significant increase, where the RL_min_ of −41.47 dB appeared at the frequency of 7.17 GHz with a match of 4.4 mm. From the 2D contour RL map of Mg_0.6_Fe_2.4_O_4_, it can also be found that the area possessing the RL value below −10 dB was also larger compared to Fe_3_O_4_ and MgFe_2_O_4_.

**Figure 8:**
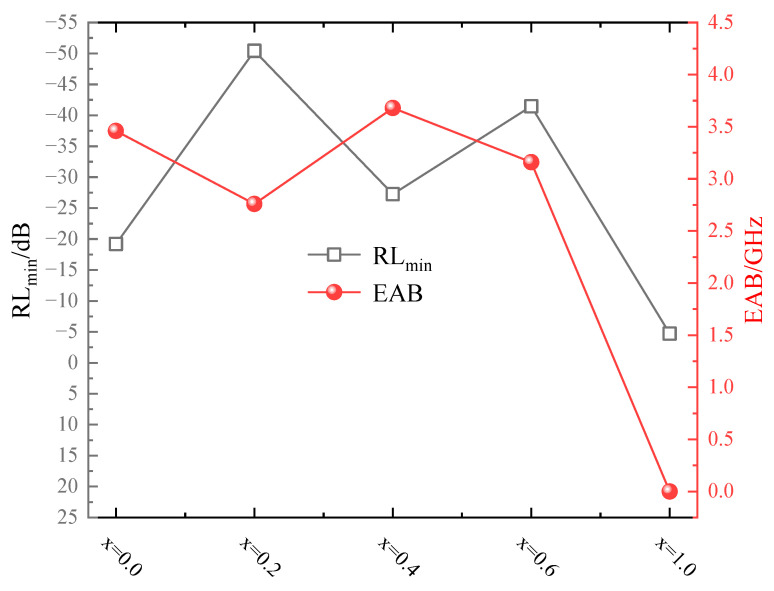
Variation curves of RL_min_ value and EAB range for Mg_x_Fe_3−x_O_4._

Figure 8 presents the variation curves of the RL_min_ value and EAB range for synthesized Mg_x_Fe_3−x_O_4_. It was obvious that the non-stoichiometric Mg^2+^-doping magnetite, such as Mg_0.2_Fe_2.8_O_4_, Mg_0.4_Fe_2.6_O_4_, Mg_0.6_Fe_2.4_O_4,_ possessed the lower RL_min_ value and wider EAB range, where the MgFe_2_O_4_ possessed the poorest microwave absorption properties. Microwave absorption performance of Fe_3_O_4_-containing materials in some previous studies was also listed in Table 3. It was obvious that the RL_min_ and EAB value of Mg_0.2_Fe_2.8_O_4_ has been superior to Fe_3_O_4_ micro or nanoscale spheres and Fe_3_O_4_-based composite material.

### 2.3. Microwave Absorption Enhancement Mechanism of Mg^2+^ for Magnetite

**Figure 9:**
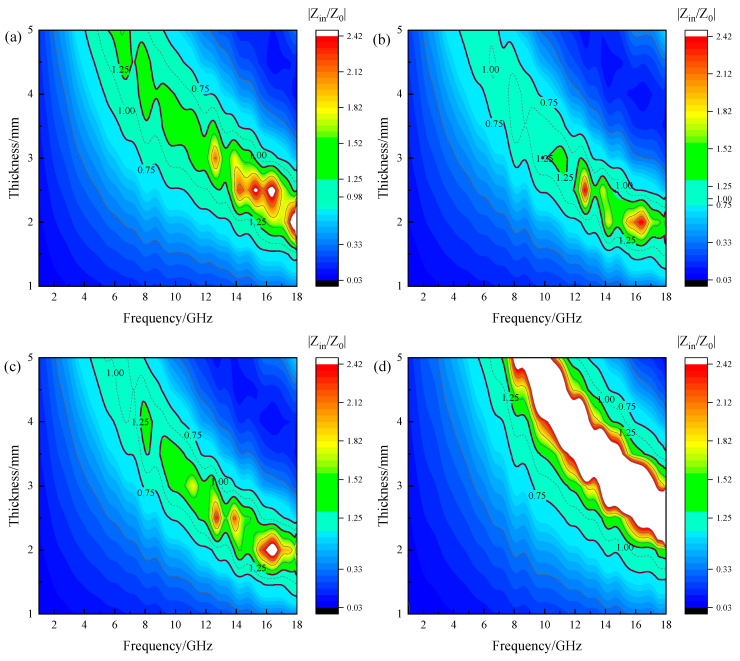
2D impedance matching plots of synthesized Mg_x_Fe_3−x_O_4_: (**a**) x = 0.0, (**b**) x = 0.2, (**c**) x = 0.6, (**d**) x = 1.0.

In order to find the enhancement mechanism of Mg^2+^ for magnetite, the impedance matching of various synthesized Mg_x_Fe_3−x_O_4_ was calculated, where the impedance matching was determined based on Equation (1). As verified, the impedance matching is a fundamental principle in the design of high-performance microwave wave absorbing materials [30,31]. When the intrinsic impedance of the absorbing material approaches that of free space (Z → 1), electromagnetic waves can propagate into the material with minimal reflection at the interface, thereby allowing the material’s intrinsic loss mechanisms (dielectric or magnetic losses, e.g.,) to effectively dissipate the electromagnetic energy. Figure 9 presents the 2D impedance matching plots of synthesized Mg_x_Fe_3−x_O_4_, where the thickness, frequency, impedance matching, and corresponding color distribution are all presented in the same standard, and the regions that approach Z → 1 (0.75 < Z < 1.25) for all the Mg_x_Fe_3−x_O_4_ were highlighted. It can be seen that the impedance matching of Mg_0.2_Fe_2.8_O_4_ demonstrated a considerably large area, as shown in Figure 7b. In comparison, the impedance matching of Fe_3_O_4_ and Mg_0.6_Fe_2.4_O_4_ both possess an area with a greater impedance mismatch in the region of relatively high frequency and low thickness, as shown in Figure 9a,c. Moreover, the impedance matching of MgFe_2_O_4_ showed the greatest impedance mismatch area, as shown in Figure 7d. Combined with the RL_min_ results as above, the reflection loss and impedance matching of Mg_x_Fe_3−x_O_4_ exhibit the same trend of variation, which provides a good explanation for the variation trend of microwave absorption performance of Mg_x_Fe_3−x_O_4_.

(1) $$Z=\left|Z_{\mathrm{in}}/Z_{0}\right|$$

**Figure 10:**
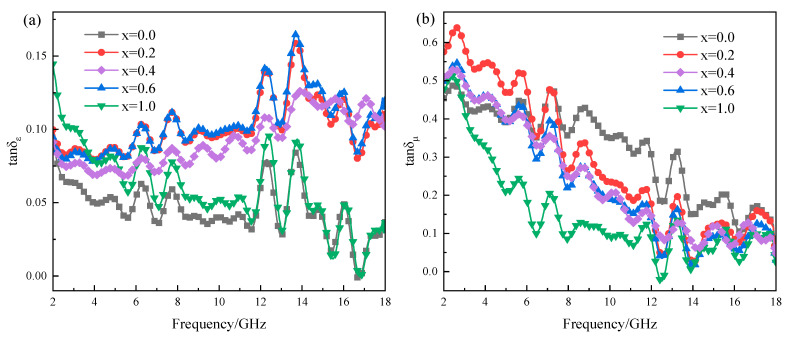
Dielectric loss tangent and magnetic loss tangent curves of Mg_x_Fe_3−x_O_4_: (**a**) dielectric loss tangent, (**b**) magnetic loss tangent.

**Figure 11:**
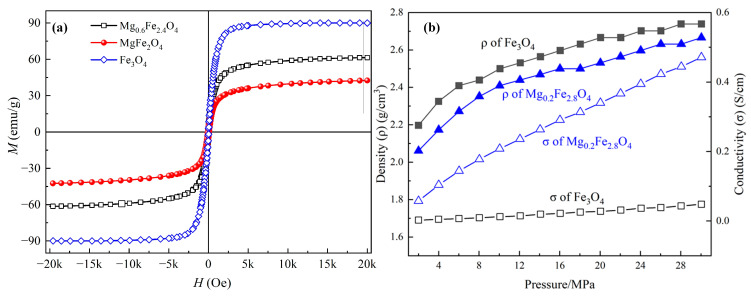
Magnetization (**a**) and conductivity (**b**) curves of Mg_x_Fe_3−x_O_4._

Dielectric and magnetic loss tangents (tanδ_ε_ = ε″/ε′, tanδ_μ_ = μ″/μ′) are also the criteria for intensifying the dielectric and magnetic losses in a material, where the contributions of the magnetic and dielectric loss mechanisms at different frequency ranges can be determined [32,33]. Figure 10 presents the dielectric loss tangent and magnetic loss tangent curves of Mg_x_Fe_3−x_O_4_. For the dielectric loss tangent as shown in Figure 10a, it was obvious that the stoichiometric Fe_3_O_4_, MgFe_2_O_4_ and three magnesium-containing magnetite solid solutions (Mg_0.2_Fe_2.8_O_4_, Mg_0.4_Fe_2.6_O_4_, Mg_0.6_Fe_2.4_O_4_) showed a completely different tendency as the function of frequency, where the variation of Fe_3_O_4_, MgFe_2_O_4_ showed a fluctuation decline tendency, inversely the magnetite solid solutions showed a rapidly rising trend, and the value of tan δ_ε_ of solid solutions were much higher than that for Fe_3_O_4_ and MgFe_2_O_4_. For the magnetic loss tangent as shown in Figure 10b, the samples showed a trend of decreasing fluctuations, where the value of Fe_3_O_4_ was a little more than the other samples. As previously verified by previous studies, an increase in tangent value could significantly reveal the decrease the reflection loss and broaden the EAB through revealing loss mechanisms and optimizing impedance matching [34,35,36]. Therefore, it can be inferred that the doping of Mg^2+^ could mainly enhance the conversion of dielectric loss to thermal energy. Figure 11a shows the magnetization curves of Fe_3_O_4_, Mg_0.6_Fe_2.4_O_4,_ and MgFe_2_O_4_. The saturation magnetization (*M*_s_) of Fe_3_O_4_, Mg_0.6_Fe_2.4_O_4_, and MgFe_2_O_4_ was 89.94 emu/g, 61.35 emu/g, and 42.43 emu/g, respectively, indicating the magnetic properties of Mg_x_Fe_3−x_O_4_ were decreased with the increase of x. Figure 11b presents the conductivity of Fe_3_O_4_ and Mg_0.2_Fe_2.8_O_4_, where it is obvious that under the same pressure and similar density, the conductivity of Mg_0.2_Fe_2.8_O_4_ is far higher than that of Fe_3_O_4_, indicating the macroscopic current and eddy currents are triggered by charge carriers, facilitating the conversion of electromagnetic energy into heat energy. On the basis of the measured results above and previous studies, the enhancement mechanism of Mg^2+^ for microwave absorption performance in Mg_x_Fe_3−x_O_4_ could be revealed. The Mg^2+^ ions could replace the Fe^3+^ ions at the A-site of magnetite, and some of the Fe^3+^ ions migrate to the B-site, which may accelerate the hopping of electrons between Fe^2+^ and Fe^3+^ to a certain extent, thus increasing the dielectric loss of magnetite [37]. Although the magnetic properties and magnetic loss of Mg_0.6_Fe_2.4_O_4_ compared to magnetite were decreased, the impedance match was optimized to a more ideal value [38].

## 3. Materials and Methods

### 3.1. Synthesis Procedure

In this work, the Mg^2+^-doping magnetite was synthesized through the reaction of Fe_2_O_3_ with MgO. For stoichiometric hematite (Fe_2_O_3_) and magnetite (Fe_3_O_4_), the thermodynamic transition temperature and atmosphere could be determined based on the Fe-O phase diagram [39]. While for non-stoichiometric hematite and magnetite solid solutions, there is no exact thermodynamic database to achieve the transformation from hematite to magnetite. Therefore, in this study, the reaction behavior for Fe_2_O_3_ with MgO under different oxygen partial pressure (*p_CO_*/(*p_CO_* + *p_CO2_*)) at 900 °C was investigated to synthesize the Mg_x_Fe_3−x_O_4_ and reveal the effect of Mg^2+^ doping on Mg_x_Fe_3−x_O_4_.

The chemical pure reagents, including Fe_2_O_3_ and MgO from China National Pharmaceutical Group Corporation, were selected according to the Fe_2_O_3_/MgO mole ratio to synthesize different content of Mg-doping magnetite solid solutions (Mg_x_Fe_3−x_O_4_, _x = 0.0, 0.2, 0.4, 0.6, 1.0_) respectively. The reagents were homogenized for 30 min, followed by pressing into the tablets with about 15 mm in diameter and 5 mm in height under a pressure of 5 MPa by the electric briquetting machine. After that, the tablets were put into a hanging basket made of Fe-Cr-Al alloy, which was heated in an in-situ thermogravimetric testing device under the specified time and temperature, followed by quenching in water. The in-situ thermogravimetric testing device consisted of an electronic balance, a resistance furnace, and a gas flow control system, where more annotations about the device have been illustrated in our previous studies [40]. The atmosphere in this work was controlled by the gas flow control system, where the gas flow velocity of N_2_ (*v*_N2_) was set as 1.0 L/min, and the sum of the flowing velocity for CO and CO_2_ was set as 1.0 L/min (*v*_CO_ + *v*_CO2_ = 1.0 L/min). The *p*_CO_/(*p*_CO_ + *p*_CO2_) was controlled by the flowing velocity ratio of CO and CO_2_ to adjust the partial oxygen pressure.

### 3.2. Characterizations

The quenched samples were first dried at 60 °C and then separated into two parts, where one part was grinded to powders below 50 μm size in the agate mortar for half an hour for powder XRD determination, where mineral phase was identified by Rigaku Ultima IV X-ray diffractometer (Rigaku Corporation, Tokyo, Japan), and Cu Kα was used as the radiation source (40 kV, 400 mA) combined with the graphite monochromator. The continuous scanning speed was 10°/min, and the data was analyzed by Crystallographica Search-Match (version of 2.1.1.0). The other part was polished for SEM and optical observations. The microstructure observation was performed by FEI Quanta 250 scanning electron microscope (FEI Corporation, Hillsboro, OR, USA). The element distribution was obtained by the XFlash 5030 EDS detector (Bruker Nano GmbH, Berlin, Germany). Magnetic properties of Mg_x_Fe_3−x_O_4_ samples were investigated at room temperature using a vibrating sample magnetometer (VSM, LakeShore, Chicago, IL, USA). The conductivity of Mg_x_Fe_3−x_O_4_ samples was measured at room temperature using a four-probe resistivity tester (ST2742C, Suzhou Lattice Electronics Co., Ltd., Suzhou, China).

In this study, Rietveld refinement was conducted to obtain the structure variation of Mg_x_Fe_3−x_O_4_. The refinement program was Fullproof (Version of July2001). The detected diffraction peaks of XRD patterns were fitted by a pseudo-Voigt profile function. The background level is simulated by the six-coefficient polynomial function. The basic crystallographic information files of Mg_x_Fe_3−x_O_4_ were obtained from the ICSD database. Subsequently, the refinement was proceeded with a sequence of scale factor, lattice parameters, zero shift, shape and asymmetry parameters, full width at half maximum (FWHM), and atomic positions, respectively.

### 3.3. Microwave Absorption Test

In this study, the microwave absorption test was also conducted to assess the microwave absorption performance of Mg_x_Fe_3−x_O_4_. Before the measurement, the Mg_x_Fe_3−x_O_4_ was first ground into powder in the agate mortar for half an hour to maintain the Mg_x_Fe_3−x_O_4_ in the same particle size level. The microwave absorption measurements were carried out using the vector network analyzer (VNA, Agilent E5071C, Santa Clara, CA, USA) equipped with a coaxial transmission waveguide, where the frequency range was 1–18 GHz. For every measurement, the Short, Open, Load, and Thru standard components are confirmed to be clean and undamaged, and the coaxial cables and connectors are inspected for any physical defects are inspected. After that, the desired frequency range, the appropriate calibration type, and the calibration kit definitions corresponding to the physical standards in use are set. Subsequently, the calibration process is performed sequentially on each port. The mixture of magnetite powders and paraffin wax at a 1:1 mass ratio, which was pressed into an appropriate toroidal-shaped sample (Φ_outer_ = 7.00 mm and Φ_inner_ = 3.04 mm) with tunable layer thickness. The VNA was calibrated in both forward and reverse directions, and the microwave source was stabilized for 1.5 h. During the normal running of VNA, the electromagnetic parameters, including *ε*′, *ε*″, *μ*′, *μ*″ of as-obtained toroidal-shaped samples were determined. The *ε*′ and *ε*″ are the real part and imaginary part of complex permittivity, whereas the *μ*′ and *μ*″ are the real part and imaginary part of complex permeability, respectively. The relative complex permittivity (*ε*_r_) and relative permeability (*μ*_r_) of the absorb medium are expressed as *ε*_r_ *= ε* − *jε*″ and *μ*_r_ *= μ*′ − *jμ*″. The reflection loss (RL) values of samples with different layer thicknesses can be calculated using MATLAB (R2021b) based on the following formulas:

(2) $$Z_{\mathrm{in}}=Z_{0}\sqrt{\mu_{r}/\varepsilon_{r}}\tanh[j(2\pi fd/c)\sqrt{\mu_{r}\varepsilon_{r}}].$$

(3) $$\mathrm{RL}(\mathrm{dB})=20\log\left|{(Z}_{\mathrm{in}}-Z_{0}{)/(Z}_{\mathrm{in}}{+Z}_{0})\right|$$

where Z_0_ represents the impedance of free space, Z_in_ is the normalized input impedance of the microwave absorption layer, *f* is the frequency of the electromagnetic wave, c is the velocity of light in free space, *d* is the layer thickness, and RL_min_ is the minimal reflection loss value. The frequency range where the RL value was below −10 dB represents that over 90% of the electromagnetic waves can be absorbed, and can be defined as the effective absorption bandwidth (EAB).

## 4. Conclusions

In this work, the magnesium-containing magnetite solid solution (Mg_x_Fe_3−x_O_4_) was synthesized, and the effect of Mg^2+^ on microwave absorption performance was investigated, whereas the microwave absorption enhancement mechanism by Mg^2+^ was also revealed.

(1) The reaction behaviors of Fe_2_O_3_ and MgO under various *p*_CO_/(*p*_CO_ + *p*_CO2_) atmosphere were investigated. It was found that Mg^2+^ could not only inhibit the re-oxidation of magnetite, but also promote the reduction of Mg_x_Fe_3−x_O_4_ to Mg_x_Fe_1−x_O, where the *p*_CO_/(*p*_CO_ + *p*_CO2_) of reduction beginning for Fe_3_O_4_, Mg_0.2_Fe_2.8_O_4_, Mg_0.4_Fe_2.6_O_4_, Mg_0.6_Fe_2.4_O_4,_ and MgFe_2_O_4_ varied from 0.22, 0.17, 0.10, 0.08, and 0.05, respectively.
(2) Microwave absorption performance of Mg_x_Fe_3−x_O_4_ for the thickness of 1–5 mm was measured and analyzed. It was found that Mg^2+^ could significantly improve the microwave absorption performance of Fe_3_O_4_, where the RL_min_ value of Mg_0.2_Fe_2.8_O_4_ has decreased to −50.43 dB compared to −19.20 dB for Fe_3_O_4_. When the content of Mg^2+^ in Fe_3_O_4_ increased to x = 1 (MgFe_2_O_4_), the performance suddenly deteriorated, where the RL_min_ value decreased to −4.72 dB.
(3) The enhancement mechanism for microwave absorption performance of Mg_x_Fe_3−x_O_4_ by Mg^2+^ was revealed through impedance matching, dielectric loss tangent, and magnetic loss and magnetization curves, where the Mg^2+^ ions could accelerate the hopping of electrons to improve the dielectric loss of magnetite, thus the impedance match could be optimized to a more ideal value.

## Figures and Tables

**Table 1:** EDS data of synthesized Mg_x_Fe_3−x_O_4_ samples.

	Position	Fe/at%	Mg/at%	O/at%	Average Data
x = 0.0	Figure 2a +1	51.44	-	48.56	Fe: 52.08 ± 1.07 at% O: 47.92 ± 1.07 at%
	Figure 2a +2	51.65	-	48.35	
	Figure 2a +3	53.15	-	46.85	
x = 0.2	Figure 2b +4	47.76	3.56	48.68	Fe: 47.81 ± 0.46 at% O: 48.68 ± 0.52 at% Mg: 3.51 ± 0.05 at%
	Figure 2b +5	48.32	3.52	48.16	
	Figure 2b +6	47.35	3.46	49.19	
x = 0.6	Figure 2c +7	43.54	10.62	45.84	Fe: 42.61 ± 0.64 at% O: 46.21 ± 0.48 at% Mg: 11.18 ± 0.56 at%
	Figure 2c +8	42.33	11.94	45.73	
	Figure 2c +9	41.97	10.98	47.06	
x = 1.0	Figure 2d +10	32.78	18.96	48.27	Fe: 33.16 ± 0.38 at% O: 48.41 ± 0.93 at% Mg: 18.43 ± 1.32 at%
	Figure 2d +11	33.41	17.11	49.48	
	Figure 2d +12	33.29	19.23	47.48	

**Table 2:** Refined structure parameters and reliability factors of synthesized Mg_x_Fe_3−x_O_4._

x	Cell Parameters (Å)			Bond Angle (°)	System	Cell Volume (Å^3^)	R_P_ (%)	R_WP_ (%)	R_exp_ (%)	Chi2
	a	b	c	α = β = γ						
0	8.398	8.398	8.398	90	cubic	592.336	2.23	3.17	2.34	1.83
0.2	8.399	8.399	8.399	90	cubic	592.445	2.02	2.57	2.33	1.21
0.4	8.406	8.406	8.406	90	cubic	593.986	2.14	2.99	2.26	1.76
0.6	8.396	8.396	8.396	90	cubic	591.806	2.19	2.88	2.39	1.45
1.0	8.395	8.395	8.395	90	cubic	591.649	2.16	2.82	2.31	1.49

**Table 3:** Microwave absorption performance of Fe_3_O_4_-containing materials in some previous studies.

Materials	Thickness (mm)	RL_min_ (dB)	EAB (GHz)	Refs.
Fe_3_O_4_ micro-spheres	4.0	−20.0	~1.9	[25]
FeSiCr@Fe_3_O_4_	3.7	−51.4	1.7	[7]
Fe_3_O_4_ nanoscale spheres	27.0	–33.5	~3.0	[26]
Fe_3_O_4_/MXene	2.5	−42.7	5.7 GHz	[27]
Fe_3_O_4_/CNTs	4.4	−51	3.9	[28]
Ti3C2TX/Fe_3_O_4_@C	1.6	−45.5	3.5 GHz	[29]
Mg_x_Fe_3−x_O_4 (x=0.2)_	4.5	−50.43	2.8 GHz	this work

## Data Availability

No new data were created, and no data are available due to privacy or ethical restrictions.

## References

[1] Liu J., Li Y., Jin C., Li Y., Yang B., Lin H. (2024). Preparation and research of high electromagnetic wave transmission type concrete in 5G frequency band. Constr. Build. Mater..

[2] Cheon S.J., Choi J.R., Lee S.B., Lee J.I., Lee H. (2024). Frequency tunable Ni–Ti-substituted Ba–M hexaferrite for efficient electromagnetic wave absorption in 8.2–75 GHz range. J. Alloy Compd..

[3] Cui Z., Yang M., Han G., Zhang H., Wang Y., Zhang Y., Li Z., He J., Yu R., Shui J. (2024). Recent advances in carbon composite films for high-performance, multifunctional and intelligent electromagnetic interference shielding and electromagnetic wave absorption. Carbon.

[4] Sharma S., Parne S.R., Panda S.S.S., Gandi S. (2024). Progress in microwave absorbing materials: A critical review. Adv. Colloid. Interfac..

[5] Jia Z., Lan D., Lin K., Qin M., Kou K., Wu G., Wu H. (2018). Progress in low-frequency microwave absorbing materials. J. Mater. Sci-Mater. El..

[6] Wang L., Wang Y., Lu J., Yan X., Liu D., Zhang X., Huang X., Wen G. (2023). Electromagnetic absorption by magnetic oxide nanomaterials: A review. ACS Appl. Nano Mater..

[7] Zhang L., Liu Y., Rehman S.U., Wang L., Chen Y., Shen S., Chen C., Liang T. (2023). In situ synthesis of Fe_3_O_4_ coated on iron-based magnetic microwave absorbing materials and the influence of oxide magnetic materials on microwave absorption mechanism. Ceram. Int..

[8] Wang X., Liu Y., Han H., Mølhave K., Sun H. (2017). Enhanced high-frequency microwave absorption of Fe_3_O_4_ architectures based on porous nanoflake. Ceram. Int..

[9] Liu X., Zhao Y., Wei Z., Zhang D. (2020). Microwave absorption enhancement of asphalt concrete with SiC-Fe_3_O_4_ mixtures modifier. Constr. Build. Mater..

[10] Bleija M., Platnieks O., Starkova O., Macutkevič J., Tsyhanok D., Orlova L., Gaidukovs S. (2024). Evaluation of thermal conductivity models and dielectric properties in metal oxide-filled poly (butylene succinate-co-adipate) composites. Sci. Rep..

[11] Zong H., Long J., Zheng J., Shen Y., Li B., Shen Y., Ren X., Lu S., Du X. (2024). Preparation of nickel slag derived Fe_3_O_4_/conductive carbon black/natural rubber composites and enhanced microwave absorption. J. Mater. Sci..

[12] Lou B., Liu Z., Sha A., Jia M., Li Y. (2020). Microwave absorption ability of steel slag and road performance of asphalt mixtures incorporating steel slag. Materials.

[13] Hu C., Li P., Zhu Y., Zhao Q., Zhang H. (2022). Experimental study on microwave absorption properties of HMA containing copper slag. Constr. Build. Mater..

[14] Wang Z., Zhang T., Zhou L. (2016). Investigation on electromagnetic and microwave absorption properties of copper slag-filled cement mortar. Cem. Concr. Comp..

[15] Chen G., Chen J., Peng J. (2015). Effects of mechanical activation on structural and microwave absorbing characteristics of high titanium slag. Powder Technol..

[16] Li K., Jiang Q., Gao L., Chen J., Peng J., Koppala S., Omran M., Chen G. (2020). Investigations on the microwave absorption properties and thermal behavior of vanadium slag: Improvement in microwave oxidation roasting for recycling vanadium and chromium. J. Hazard. Mater..

[17] Harrison R.J., Putnis A. (1996). Magnetic properties of the magnetite-spinel solid solution: Curie temperatures, magnetic susceptibilities, and cation ordering. Am. Mineral..

[18] Higuchi K., Tanaka T., Sato T. (2007). Reaction behavior of dolomite accompanied with formation of magnetite solid solution in iron ore sintering process. ISIJ Int..

[19] Li Y., Jin Y., Raza H., Wang Y., Chen Q., Zou X., Ren Z., Guo J., Zheng G., Cheng J. (2025). Dual driving strategy from micro-polarization to macroscopic conductance: Tailoring optimized low-frequency and wide-band microwave absorption in high-entropy oxides. J. Mater. Sci. Technol..

[20] Zhang Y., Huang C., Wang D., Ye F., Lu F., Hu C., Cheng L. (2025). Effect of Sm doping on structure and microwave absorption properties of brownmillerite oxide Ca_2_Fe_2_O_5_. Ceram. Int..

[21] Jiang W., Xu S., Lv C., Lan D., Zhang S., Gao Z., Jia Z., Wu G. (2025). Multi-scale Engineering of N-doped Carbon Nanofibers with Co_3_O_4_/CeO_2_ Heterostructures: Tailoring Heterointerface Polarization for Microwave Absorption. Carbon.

[22] Jastrzębska I., Przystaś J., Pająk O., Błachuta T., Drożdż P., Mandal S. (2025). Corrosion and wettability of Magnesium Orthotitanate (Mg_2_TiO_4_) refractory aggregate by copper slags with varying iron/silica ratios. J. Eur. Ceram. Soc..

[23] Ren G., Wang X., Zheng B., Zhang Z., Yang L., Yang X. (2020). Fabrication of Mg doped magnetite nanoparticles by recycling of titanium slag and their application of arsenate adsorption. J. Clean. Prod..

[24] Sun L., Wu J., Wang J., Yang Y., Xu M., Liu J., Yang C., Cai Y., He H., Du Y. (2022). In-situ constructing nanostructured magnesium ferrite on steel slag for Cr (VI) photoreduction. J. Hazard. Mater..

[25] Ni S., Sun X., Wang X., Zhou G., Yang F., Wang J., He D. (2010). Low temperature synthesis of Fe_3_O_4_ micro-spheres and its microwave absorption properties. Mater. Chem. Phys..

[26] Li L.G., Lai Q., Zeng G.X., Li Y.J., Xie H.Z., Kwan A.K.H. (2023). Combined effects of micro and nano Fe_3_O_4_ on workability, strength, packing, microstructure and EM wave absorbing properties of mortar. Constr. Build. Mater..

[27] Xu J., Tang S., Liu D., Bai Z., Xie X., Tian X., Xu W., Hou W., Meng X., Yang N. (2022). Rational design of hollow Fe_3_O_4_ microspheres on Ti_3_C_2_T_x_ MXene nanosheets as highly-efficient and lightweight electromagnetic absorbers. Ceram. Int..

[28] Zhu L., Zeng X., Chen M., Yu R. (2017). Controllable permittivity in 3D Fe_3_O_4_/CNTs network for remarkable microwave absorption performances. RSC Adv..

[29] Deng B., Wang L., Xiang Z., Liu Z., Pan F., Lu W. (2021). Rational construction of MXene/Ferrite@C hybrids with improved impedance matching for high-performance electromagnetic absorption applications. Mater. Lett..

[30] Jazirehpour M., Ebrahimi S.S. (2015). Effect of aspect ratio on dielectric, magnetic, percolative and microwave absorption properties of magnetite nanoparticles. J. Alloys Compd..

[31] Xu H., Deng J., Bai Z., Zhao B., Wang G., Yang L. (2023). Natural magnetite/coke composite: A novel promising microwave absorption material. J. Alloys Compd..

[32] Cao Y., Liu C., Xue Z., Jiang T., Fang G., Peng K., Zhang Y. (2022). Excellent microwave absorption of Fe_3_O_4_/Ag composites attained by synergy of considerable magnetic loss and dielectric loss. Ceram. Int..

[33] Jadav M., Bhatnagar S.P. (2020). Particle size controlled magnetic loss in magnetite nanoparticles in RF-microwave region. IEEE Trans. Magn..

[34] Hussain A., Gul I.H., Khan M.Z. (2025). Enhancement of dielectric, magnetic and microwave absorption properties of Co^2+^-Zr^4+^ substituted SrFe_12_O_19_ nanoparticles. Ceram. Int..

[35] Huang X., Qiao M., Lu X., Li Y., Ma Y., Kang B., Quan B., Ji G. (2021). Evolution of dielectric loss-dominated electromagnetic patterns in magnetic absorbers for enhanced microwave absorption performances. Nano Res..

[36] Zhu S., Guo X., Xiang R., Cheng L., Ye F., Li Z., Yao Q., Qi H., Long Q. (2025). Balance dielectric and magnetic losses to enhance the microwave absorption performance of CaFe_0. 5_Mn_0. 5_O_3−δ_@ Co_3_O_4_@ Fe_3_O_4_. J. Alloys Compd..

[37] Singh N., Agarwal A., Sanghi S., Singh P. (2011). Effect of magnesium substitution on dielectric and magnetic properties of Ni–Zn ferrite. Physica. B.

[38] Abbas S.M., Dixit A.K., Chatterjee R., Goel T.C. (2007). Complex permittivity, complex permeability and microwave absorption properties of ferrite–polymer composites. J. Magn. Magn. Mater..

[39] Sundman B. (1991). An assessment of the Fe-O system. J. Phase Equilibria.

[40] Du Y., Guo X.M. (2024). Displacement and Migration Behavior of Al^3+^ in Ca_2_Fe_2−x_Al_x_O_5_ Solid Solution During Reduction Process. Metall. Mater. Trans. B.

